# Regional structural abnormalities in thalamus in idiopathic cervical dystonia

**DOI:** 10.1186/s12883-024-03680-6

**Published:** 2024-05-24

**Authors:** Yuhan Luo, Huiming Liu, Linchang Zhong, Ai Weng, Zhengkun Yang, Yue Zhang, Jiana Zhang, Xiuye He, Zilin Ou, Zhicong Yan, Qinxiu Cheng, Xinxin Fan, Xiaodong Zhang, Weixi Zhang, Qingmao Hu, Kangqiang Peng, Gang Liu, Jinping Xu

**Affiliations:** 1 Department of Neurology, Guangdong Provincial Key Laboratory for Diagnosis and Treatment of Major Neurological Diseases, National Key Clinical Department and Key Discipline of Neurology, The First Affiliated Hospital, Sun Yat-sen University, Guangzhou, 510080 China; 2 Department of Medical Imaging, State Key Laboratory of Oncology in Southern China, Collaborative Innovation Center for Cancer Medicine, Sun Yat-Sen University Cancer Center, Guangzhou, 510060 China; 3 Institute of Biomedical and Health Engineering, Shenzhen Institutes of Advanced Technology, Chinese Academy of Sciences, Shenzhen, 518055 China

**Keywords:** Atrophy, Grey matter volume, Idiopathic cervical dystonia, Structural magnetic resonance imaging, Thalamic nuclei

## Abstract

**Background:**

The thalamus has a central role in the pathophysiology of idiopathic cervical dystonia (iCD); however, the nature of alterations occurring within this structure remain largely elusive. Using a structural magnetic resonance imaging (MRI) approach, we examined whether abnormalities differ across thalamic subregions/nuclei in patients with iCD.

**Methods:**

Structural MRI data were collected from 37 patients with iCD and 37 healthy controls (HCs). Automatic parcellation of 25 thalamic nuclei in each hemisphere was performed based on the FreeSurfer program. Differences in thalamic nuclei volumes between groups and their relationships with clinical information were analysed in patients with iCD.

**Results:**

Compared to HCs, a significant reduction in thalamic nuclei volume primarily in central medial, centromedian, lateral geniculate, medial geniculate, medial ventral, paracentral, parafascicular, paratenial, and ventromedial nuclei was found in patients with iCD (*P <* 0.05, false discovery rate corrected). However, no statistically significant correlations were observed between altered thalamic nuclei volumes and clinical characteristics in iCD group.

**Conclusion:**

This study highlights the neurobiological mechanisms of iCD related to thalamic volume changes.

## Background

Idiopathic cervical dystonia (iCD), the most common form of adult-onset dystonia, is characterised by sustained or intermittent neck movements caused by involuntary muscle contractions, resulting in abnormal movements and postures of the head, neck, and/or shoulders. In addition, patients with iCD may also exhibit nonmotor symptoms such as mood disorders, pain, cognitive deficits, and sleep disorders, together with motor manifestations that influence daily living activities and reduce the quality of life in these patients [1]. However, the underlying cause and pathophysiology of iCD remain incompletely understood.

Current neurophysiological and neuroimaging evidence shows a network model in which various brain regions play a specific role in the iCD pathogenesis, including the basal ganglia, thalamus, cerebellum, and sensorimotor cortex [2, 3]. The thalamus is a key integrative hub in this network, receiving and distributing information among different brain regions [4]. Apart from being a simple transponder, the thalamus contributes to signal processing within cortical hierarchies, involving regulation of emotions, arousal, cognition, wakefulness, motor control, and sensory information processing [5, 6]. Lesion studies have showed that thalamic lesions are prone to induce CD [7, 8], further emphasizing the potential key role of the thalamus in the iCD. Previous functional neuroimaging studies have reported increased cortical activation [9], increased regional spontaneous brain activity [10], glucose hyper-metabolism [11], and altered functional connectivity profiles [10] in the thalamus as well as abnormal cerebellar-basal ganglia-thalamo-cortical sensorimotor circuit [12] in patients with iCD. Structural neuroimaging studies also showed brain structural abnormalities in patients with iCD, but these often yielded diverse results. Either an increase [3, 13] or a decrease [14, 15] in thalamic volume has been reported in patients with iCD compared to healthy controls (HCs). Additionally, two neuroimaging studies investigating white matter microstructural abnormalities have identified increased fractional anisotropy in the bilateral thalamus in patients with iCD [13, 16]. In these studies, the thalamus was considered as a single, homogeneous structure, disregarding potentially useful information about distinct thalamic nuclei. The thalamus contains distinct nuclei serving different functions, which may be related to different symptoms or disorders [17, 18]. However, to our knowledge, no study has explored structural alterations in the distinct thalamic nuclei and their relationship to the motor and nonmotor manifestations in patients with iCD. Recently, a statistical atlas of the thalamus was constructed using ultra-high-resolution ex vivo magnetic resonance imaging (MRI) combined with in vivo data [19] (available in FreeSurfer 7, https://surfer.nmr.mgh.harvard.edu/fswiki/rel7downloads). This tool enables the accurate measurement of each individual thalamic nuclei volume, exhibiting robust agreement with histological findings and showing excellent test-retest reliability [19]. This tool has recently been successfully employed to examine region-specific thalamic alterations in neurological disorders, including Parkinson’s disease [20], restless legs syndrome [21], and epilepsy [18]. Therefore, it is an ideal atlas to investigate whether and how thalamic nuclear volume change occurs in iCD.

In this study, we analysed structural MRI data from 37 patients with iCD and 37 HCs to examine (1) whether grey matter-related abnormalities are only restricted to specific thalamic subregions and (2) whether and how alterations in volumes of thalamic subregions are associated with motor or nonmotor symptoms in patients with iCD. We hypothesised that there is heterogeneity in the morphological changes occurring within distinct thalamic nuclei in patients with iCD.

## Methods

### Participants

Patients were recruited from our outpatient clinic for movement disorders between April 2019 and July 2023. The diagnosis of iCD was made by two senior neurologists based on the standard criteria [1, 22]. Patients were excluded if they (i) had dystonia involving other body sites in addition to neck muscles; (ii) reported evidence of stroke, Parkinson’s disease, Alzheimer’s disease, epilepsy, and traumatic brain injury; (iii) had a family history of movement disorders as well as a history of exposure to antipsychotic drugs before the onset of dystonia; (iv) had any conditions that contradicted with cerebral MRI; (v) received botulinum toxin (BoNT) injections within 3 months and oral medications for approximately 24 h before MRI scans. HCs were recruited using the same exclusion criteria.

### Clinical measurements

The demographics and clinical characteristics, including the participants’ age, gender, education level, handedness, duration of disease, duration of BoNT injections, and times of BoNT injections, were collected via in-person interviews before MRI scanning [23]. The Toronto Western Spasmodic Torticollis Rating Scale (TWSTRS) [24], consisting of three subscales assessing motor severity, disability, and pain, was employed to evaluate symptom severity in patients with iCD by a trained neurologist. Furthermore, the Hamilton anxiety rating scale (HAMA) [25], Hamilton depression rating scale (HAMD) [26], and Mini-Mental State Examination (MMSE) were also conducted before MRI scanning to evaluate patients’ mood and cognitive function.

### Image acquisition

Three-dimensional T1-weighted data were collected using a 3T MRI scanner (Tim Trio; Siemens, Erlangen, Germany) with a magnetisation-prepared rapid-acquisition gradient-echo pulse sequence. The main parameters were repetition time = 2530 ms; echo time = 4.45 ms; inversion time = 1100 ms; flip angle = 7°; matrix dimensions = 256 mm × 256 mm; voxel size = 1 × 1 × 1 mm^3^; and 192 slices.

### Image preprocessing

**Fig. 1:**
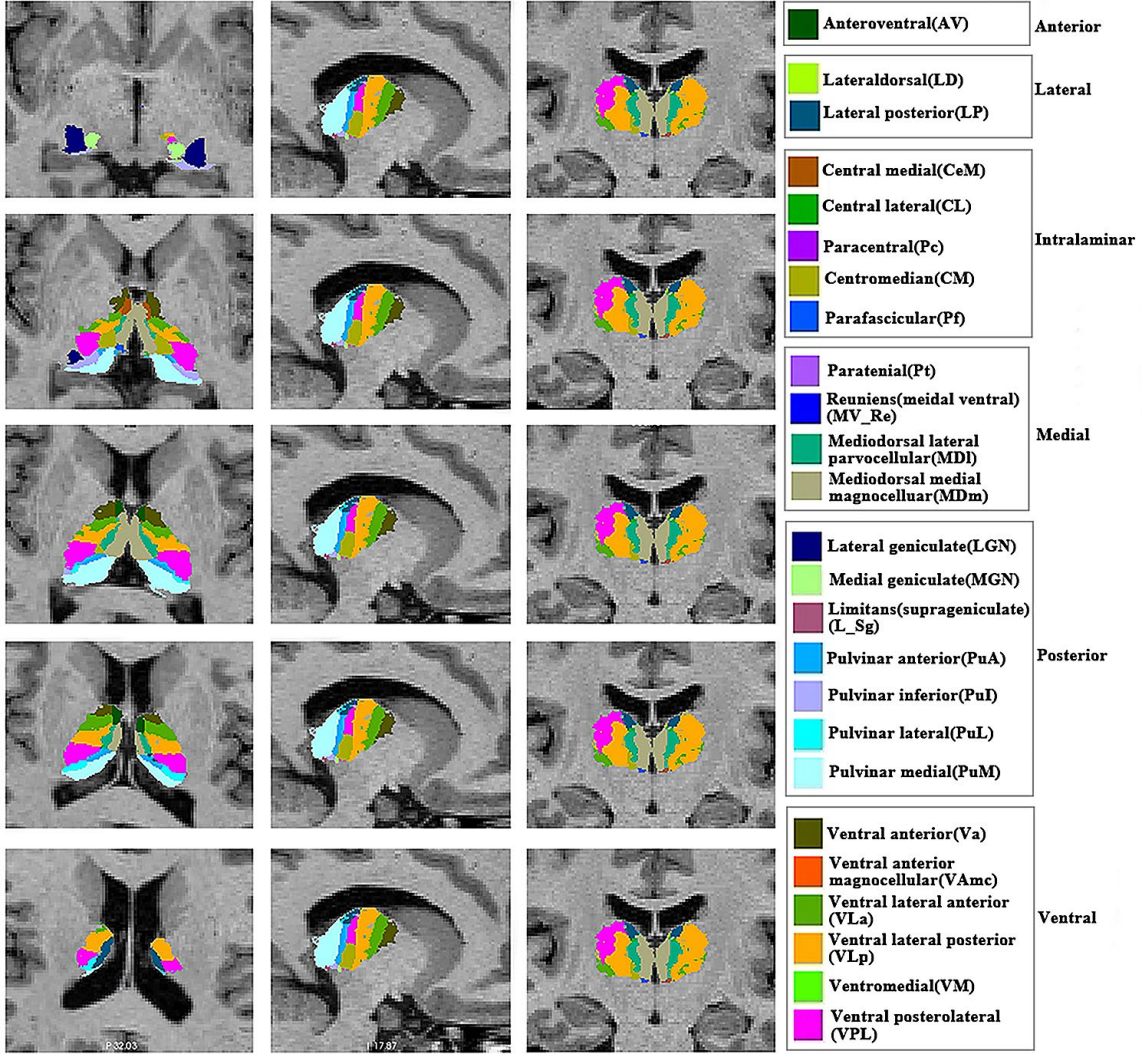
The thalamic nucleus in one healthy participant. The images are shown using Freeview (https://surfer.nmr.mgh.harvard.edu/fswiki/FreeviewGuide).

All T1 images were processed using the standard segmentation pipeline in FreeSurfer v7.1.1 with default settings (https://surfer.nmr.mgh.harvard.edu). The main steps included skull stripping, Talairach registration and initialisation of cortical surface reconstruction, cortical atlas registration, and subcortical parcellation. We implemented automatic parcellation of 25 thalamic nuclei in each hemisphere based on manual delineation combining in vivo and ex vivo data to quantify thalamic nuclei volumes [19]. These nuclei included anteroventral, laterodorsal and lateral posterior, ventral anterior, ventral anterior magnocellular, ventral lateral anterior, ventral lateral posterior (VLp), ventral posterolateral (VPL), and ventromedial (VM), central medial (CeM), central lateral, paracentral (Pc), centromedian (CM), and parafascicular (Pf), paratenial (Pt), Reuniens (medial ventral) (MV(Re)), mediodorsal medial magnocellular, and mediodorsal lateral parvocellular, lateral geniculate (LGN), medial geniculate (MGN), suprageniculate, pulvinar anterior, pulvinar medial, pulvinar lateral, and pulvinar inferior (Fig. 1). Visualised inspection confirmed that the automatic segmentation and labelling were performed accurately. Finally, Freeview (https://surfer.nmr.mgh.harvard.edu/fswiki/FreeviewGuide/FreeviewIntroduction) was used to show the thalamic nuclei.

### Calculation of thalamic nuclei volumes

Between-group differences of grey matter volume in the thalamic nuclei were performed using a general linear model with age, gender, and estimated total intracranial volume as covariates. Finally, the results were corrected by a false discovery rate (FDR) with *P* < 0.05.

### Correlation analyses

Correlations between abnormal thalamic nuclei volumes and TWSTRS severity scores, disability scores, pain scores, disease durations, as well as HAMA and HAMD scores in patients with iCD were performed using partial correlations with age, gender, and estimated total intracranial volume as covariates. Meanwhile, correlations between HAMA/HAMD scores and TWSTRS severity scores, disability scores, pain scores, and disease durations in patients with iCD were also analysed using partial correlations with age and gender as covariates. Statistical significance was set as *P* < 0.05.

### Statistical analyses

Age, estimated total intracranial volume, HAMA scores, HAMD scores, and MMSE scores were compared using two sample *t*-tests or Mann–Whitney *U* tests after normality testing using the Shapiro–Wilk test. Pearson *χ*^*2*^ test was performed for gender comparison. All analyses were performed using the Statistical Package for the Social Sciences (SPSS) version 25.0 (SPSS Inc., Chicago, IL).

## Results

### Demographic information and clinical characteristics

Overall, 37 patients with iCD and 37 HCs were included in this study. However, the TWSTRS, HAMD, and MMSE scores were completed by 29, 36, and 35 patients with iCD, respectively. The primary reason for incomplete clinical evaluations in some of the patients was their discomfort experienced from abnormal movements and postures, leading to their refusal to cooperate with long-term scale assessment. The clinical and demographic information of the 37 patients with iCD and 37 HCs are presented in Table 1. The iCD and HCs groups did not differ in gender, estimated total intracranial volume, and MMSE scores. There were significant differences in age, HAMA, and HAMD scores between patients with iCD and HCs.

**Table 1:** Participants’ demographics and clinical characteristics

	iCD (n = 37)		HCs (n = 37)	*P*
Gender (Female/Male)		17/20	24/13	0.102
Median age, years (range)		39 (19–66)	47 (33–55)	0.002
eTIV, cm^3^ (range)		1459 (935–1787)	1332 (924–1883)	0.182
Median MMSE scores (range)		28 (25–30)^a^	29 (25–30)	0.221
Median HAMA scores (range)		6 (0–23)	1 (0–8)	< 0.001
Median HAMD scores (range)		5 (0–18)^b^	1 (0–7)	< 0.001
Disease duration, years (range)		1.5 (0.12-13)	-	-
BoNT injections (yes/no)		14/23	-	-
Median TWSTRS severity scores (range)		18 (7–25)^c^	-	--
Median TWSTRS disability scores (range)		10 (0–23)^c^	-	-
Median TWSTRS pain scores (range)		5.5 (0–14)^c^	-	-

*Note*^a^ only 35 participants are available. ^b^ only 36 participants are available. ^c^ only 29 participants are available. *Abbreviations* BoNT, Botulinum toxin; eTIV, estimated total intracranial volume; iCD, idiopathic cervical dystonia; HAMA, Hamilton anxiety rating scale; HAMD, Hamilton depression rating scale; HCs, healthy controls; MMSE, Mini-Mental State Examination; and TWSTRS, Toronto Western Spasmodic Torticollis Rating Scale

### Differences of the thalamic nuclei volumes

**Fig. 2:**
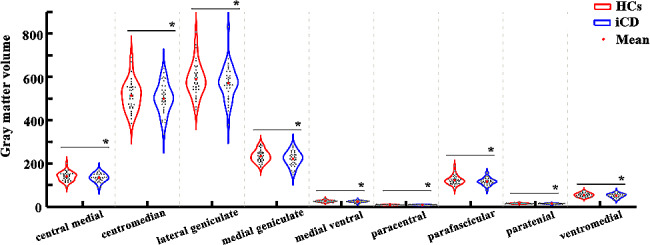
Differences in the thalamic nuclei volumes. Thalamic nuclei’s mean grey matter volumes are compared between idiopathic cervical dystonia and healthy controls using a general linear model with age, gender, and estimated total intracranial volume as covariates. The results are corrected by false discovery rate (FDR) with *P* < 0.05. *represents significant difference. Abbreviations: iCD, idiopathic cervical dystonia, and HCs, healthy controls. The abbreviations of thalamic nuclei are listed in Table 2

Patients with iCD compared to HCs showed significant volume atrophy mainly in the CeM, CM, LGN, MGN, MV(Re), Pc, Pf, Pt, and VM nuclei (*P* < 0.05, FDR corrected; Fig. 2 and Table 2). Trends for volume reduction of VLp (*P* = 0.053, FDR corrected) and VPL (*P* = 0.057, FDR corrected) nuclei were also observed in patients with iCD relative to HCs (Table 2).

**Table 2:** Differences of thalamic nuclei volumes between idiopathic cervical dystonia and healthy controls

Thalamic Nucleus	Abbreviations	HCs	iCD	Corrected *P*
Anteroventral	AV	271.17 ± 39.60	269.45 ± 34.34	0.161
Central medial	CeM	139.95 ± 20.46	134.94 ± 18.82	0.032*
Central lateral	CL	70.09 ± 18.39	68.66 ± 12.30	0.216
Centromedian	CM	513.31 ± 68.61	499.73 ± 68.65	0.044*
Lateraldorsal	LD	51.03 ± 15.91	47.80 ± 12.75	0.208
Lateral geniculate	LGN	589.09 ± 76.65	571.24 ± 79.58	0.032*
Lateral posterior	LP	240.39 ± 46.78	245.27 ± 29.81	0.566
Limitans (suprageniculate)	L-Sg	49.47 ± 7.617	46.44 ± 10.79	0.208
Mediodorsal lateral parvocellular	MDl	522.70 ± 46.75	518.38 ± 51.52	0.126
Mediodorsal medial magnocelluar	MDm	1500.73 ± 161.42	1491.45 ± 163.96	0.092
Medial geniculate	MGN	234.22 ± 27.30	221.81 ± 32.96	0.045*
Reuniens (medial ventral)	MV(Re)	25.54 ± 4.39	23.95 ± 4.70	0.032*
Paracentral	Pc	8.55 ± 1.17	8.41 ± 1.07	0.046*
Parafascicular	Pf	122.30 ± 19.79	116.67 ± 16.71	0.032*
Paratenial	Pt	14.97 ± 1.95	14.47 ± 1.88	0.044*
Pulvinar anterior	PuA	439.48 ± 38.65	452.30 ± 46.04	0.385
Pulvinar inferior	PuI	521.98 ± 73.14	552.26 ± 83.20	0.658
Pulvinar lateral	PuL	377.68 ± 46.59	398.42 ± 59.43	0.658
Pulvinar medial	PuM	2268.23 ± 254.50	2359.87 ± 260.96	0.458
Ventral anterior	VA	805.43 ± 89.78	817.68 ± 88.41	0.053
Ventral anterior magnocellular	VAmc	63.33 ± 8.19	64.18 ± 7.74	0.053
Ventral lateral anterior	VLa	1258.57 ± 146.47	1261.48 ± 138.49	0.053
Ventral lateral posterior	VLp	1659.83 ± 206.78	1652.35 ± 195.23	0.053
Ventromedial	VM	54.70 ± 8.80	51.63 ± 9.77	0.044*
Ventral posterolateral	VPL	1918.61 ± 250.33	1879.88 ± 261.50	0.057

*Note* * represents significant results after false discovery rate (FDR) corrected with *P* < 0.05. *Abbreviations* iCD, idiopathic cervical dystonia, and HCs, healthy controls

### Correlational analyses

No statistically significant correlations were observed between altered thalamic nuclei volumes and TWSTRS severity scores, disability scores, pain scores, disease durations, HAMA scores, and HAMD scores in patients with iCD after adjusting for age, gender and estimated total intracranial volume as covariates. Moreover, no statistically significant correlations were observed between HAMA/HAMD scores, TWSTRS scores, and disease durations in patients with iCD with age and gender as covariates.

## Discussion

In this study, we observed a significant decrease in grey matter volume of specific thalamic nuclei, including CeM, CM, LGN, MGN, MV(Re), Pc, Pf, Pt, and VM in patients with iCD compared to HCs. However, no statistically significant correlations were found between these thalamic nuclei volumes and clinical characteristics in iCD group. These results support the hypothesis that regional thalamic atrophy is present in patients with iCD.

The intralaminar thalamic nuclei are the primary source of excitatory input from the thalamus to the striatum [27]. The caudal group of intralaminar thalamic nuclei, namely the CM and Pf nuclei, exhibit extensive and specific connections with the basal ganglia and motor cortex, indicating potential involvement of the CM-Pf complex in motor functions. Projections from the CM and Pf are principally extended to the striatum, which have been shown to mediate a reciprocal thalamostriatal interaction that plays an important role in both normal and pathological movement [6, 28, 29]. Semenova and his colleagues discovered that movement-sensitive CM-Pf neurons exhibit selective sensitivity towards voluntary neck and hand movements in patients with iCD [30]. The most pronounced and prolonged responses were observed during movements involving neck muscles and involuntary dystonic movements, indicating the participation of the CM-Pf complex in motor behaviour and its potential involvement in the pathophysiology of iCD. However, because of the absence of a control group in this study, whether these findings are directly relevant to the disease’s pathophysiology remains uncertain [30]. Our findings regarding CM and Pf nuclei atrophy found in patients with iCD may provide further evidence for their involvement in the pathophysiology of iCD. Projections from intralaminar nuclei transmit sensory signals to striatal cholinergic interneurons, eliciting a lasting pause after a burst of spikes and facilitating the integration of cortical inputs with medium spiny neurons, thereby playing a crucial role in motor function [31]. A study on mice carrying the DYT1 dystonia mutation demonstrated that an altered thalamostriatal input pattern leads to abnormal cholinergic signalling, disrupting the integration between corticostriatal and thalamostriatal, which might result in an altered motor output and predispose DYT1 gene mutation carriers to develop dystonic movements [32]. Furthermore, the thalamic rostral intralaminar nuclei, including the CeM and Pc nuclei, contribute to a range of behaviours such as sensorimotor coordination, pain modulation, cognition, and arousal processing through extensive projections to the striatum and cortex [6, 33]. Among these nuclei, Pc contributes to motor control and processes pain signals conveyed through the spinoparabrachial pathway [34–36]. Sensory symptom, such as pain, is commonly reported in patients with iCD [1]; however, the underlying pathophysiological mechanism of this nonmotor manifestation remains unclear. It is reasonable to speculate that such symptom might be caused by the dysfunction of Pc nucleus in iCD. In functional MRI studies, iCD has been considered a consequence of an abnormal basal ganglia-thalamo-cortical circuit [12]. Therefore, the present findings might improve our understanding of the role of underlying structural substrates in abnormal functional brain states in iCD.

The exploration of ventral thalamic nuclei is particularly interesting because of their involvement in sensorimotor information processing and being one of the major targets for surgical treatment of movement disorders [37, 38]. It has been reported that four patients with iCD received markedly improved dystonic head tremor and dystonia after continuous bilateral thalamic ventral lateral anterior nucleus stimulation for 3 months [38]. Previous studies have identified specific patterns of neuronal activity and pathological features of the ventral thalamus in iCD [39, 40]. Ventral thalamic neurons exhibiting dystonia-frequency activity in patients with iCD and suppression of this activity induced immediate improvement and subsequent further enhancement of dystonic movement and posture, revealing the critical role of the ventral thalamus as a key node within the network associated with dystonia [39, 40]. In our study, we observed that patients with iCD exhibited atrophy in the thalamus’s VM, VLp, and VPL nuclei. However, the latter two nuclei differences between groups did not reach statistical significance via FDR correction. VM and VLp are known as the motor thalamus, connecting the motor areas of the cerebral cortex to the basal ganglia and cerebellum [41]. VPL, as a part of the somatosensory thalamus, receives neuronal input from the medial lemniscus and spinothalamic tracts and subsequently projects to the somatosensory cortex [42]. Precise motor control relies on optimal processing of afferent inputs from various sensory systems, such as visual and somatosensory (e.g., touch and proprioception). However, abnormalities in the integration of sensory input with control of motor output in focal dystonia have been highlighted by recent neurophysiological studies [43–46]. Sensory-motor integration involves a complex cerebral network that includes the basal ganglia-thalamic-frontal cortex loop as well as the parietal cortex and cerebellum. The neural degeneration of different levels within this network has been reported to be associated with deficits in sensory-motor integration observed in dystonia [43, 45]. Therefore, the impaired sensory-motor integration may be partly explained by atrophy in these subregions of the thalamus in patients with iCD. Notably, the fundamental role of somatosensory deficits in the complex process of sensory-motor integration in iCD is now widely recognised, especially that related to proprioception. Two studies based on vibration over the neck muscles in patients with iCD have showed that entrainment of proprioceptive afferent pathways induces dystonic activity, providing evidence for a causal relationship between proprioceptive afferents and motor symptoms observed in iCD [47, 48]. Another study evaluating somatosensory temporal discrimination threshold (STDT) values in patients with focal dystonia reported that movement execution led to a greater and longer-lasting increase in STDT values in focal hand dystonia (FHD) and CD but not in blepharospasm (BSP). These results suggest that the motor overflow underpinning dystonic posture and proprioceptive afferent signalling from the forearm (in the FHD) and neck muscles (in the CD) may alter sensory gating of tactile information when reaching the thalamic level. This abnormality also indicates that sensory gating mechanisms induced by movement execution vary among the sub-types of dystonia [49]. In addition, BoNT-A demonstrated a significant reduction in the abnormally higher STDT modulation during movement execution in patients with CD and FHD while being normal in BSP. One potential mechanism on how BoNT-A improves motor symptoms may involve its indirect central effects, which modulate the overflow of proprioceptive signals from dystonic muscles to the thalamus [50, 51]. Previous research has suggested that proprioceptive signals originating from facial muscles project to the thalamic ventral posteromedial nucleus, whereas those from neck muscles are thought to project to the VPL nucleus [52–54]. Moreover, the somatotopic representation of body parts in different brain regions constitutes one of the neural bases for the heterogeneity of different dystonia types [55]. Based on these aforementioned studies and our findings regarding nuclear atrophy in VPL, it is hypothesised that VPL may be specifically involved in abnormal processing of proprioceptive signals in patients with iCD. Future research should focus on investigating regional structural and functional changes in VPL and its connectivity with other brain regions using multimodal MRI techniques with larger sample sizes in iCD. Studying how proprioception modulation affects VPL neuronal activity patterns in iCD would also be valuable [56].

Our study also revealed a higher prevalence of anxiety (37.8%) and depressive symptoms (36.1%) in patients with iCD compared to HCs. Furthermore, no significant correlations were found between HAMA/HAMD scores and the severity of motor symptoms or disease duration in patients with iCD, demonstrating that mood disorders are not simply a consequence of the movement disorder. Consistent with our results, higher rates of all psychiatric comorbidities were observed in those with iCD, with depression and anxiety being the most commonly diagnosed conditions. Additionally, anxiety and depressive symptoms were reported to precede the onset of iCD generally and reach a peak 12 months before dystonia diagnosis, indicating that both psychiatric disorders are either prodromal symptoms or reflect shared aetiological mechanisms [57–59]. However, research on the mechanisms underlying anxiety or depressive symptoms in iCD is relatively limited. Current studies propose that dysfunction within basal ganglia-thalamo-cortical circuits underlies motor and psychiatric manifestations in iCD [60, 61]. In line with this hypothesis, growing evidence shows that the thalamus is one of the core dysconnectivity nodes in anxiety and depression [62, 63]. The Re nucleus, a thalamic midline nucleus, exhibits reciprocal connections with the prefrontal cortex and hippocampus, serving as key intermediaries between these structures to regulate emotional behaviours [64]. Previous animal studies have indicated the involvement of Re nucleus dysfunction in anxiety and depressive-like behaviour, which may contribute to the amalgamation of symptoms commonly observed in mental disorders such as anxiety and depression [65, 66]. Interestingly, our study also identified atrophy in the Re nucleus in patients with iCD. Therefore, we speculate that volumetric changes in this specific thalamic region may be associated with mood disorders observed in iCD. A voxel-based morphometry study investigating another type of cranial dystonia with and without depression partially supported our hypothesis by revealing volume reduction in the frontal cortex and hippocampus in the depression group. However, this study did not specifically examine morphometric differences within thalamic nuclei [67]. Further research is needed to understand the underlying pathophysiology of anxiety and depression in patients with iCD.

Furthermore, we did not find correlations between atrophy in these thalamic nuclei and the severity of motor symptoms in patients with iCD, which appears to contradict the current view that implicates the involvement of the cerebello-thalamo-cortical circuit in symptom severity across different types of focal dystonia [68, 69]. A plausible explanation could be that while a potential mechanism involves a similar network model responsible for focal dystonia, different types of dystonia originate from dysfunction in one of these regions before impacting the network more extensively. In other words, diverse forms of focal dystonia might emerge at different levels within the network, resulting in nodes with varying hierarchies of influence and specific roles. Only structural abnormalities in high-order nodes might determine the severity of symptoms in patients with iCD [55]. In line with our results, previous neuroimaging studies on patients with iCD also reported a lack of correlations between reduced thalamic volume and symptom severity [14, 15]. Moreover, another study demonstrated that only thalamic connections to other brain regions were altered when symptoms improved after BoNT treatment in iCD, without affecting its loss of responsiveness [70]. Additionally, patients with unavailable TWSTRS scores might potentially influence the correlations between those and changes in thalamic nuclei volume. Further investigation is needed to understand the role of specific thalamic nuclei within the network model of iCD.

This study has several limitations. First, our sample size is relatively small, which may limit the statistical power to detect significant differences in VLp and VPL nuclei volumes between groups after FDR corrections and correlations between atrophy in thalamic nuclei and severity in motor and nonmotor symptoms in patients with iCD. Second, the iCD and HCs groups are not matched for age, although we have regressed age and intracranial volume as covariates when comparing differences of the thalamic nuclear volumes between the two groups to mitigate its effect. Third, some patients’ lack of TWSTRS scores might influence the correlations between those and altered thalamic nuclei volume. Finally, some previous studies have demonstrated that BoNT injections can induce alterations in subcortical white matter microstructure [71] and sensorimotor network activation [72] in patients with iCD. The effects of BoNT on the thalamic nuclei volumes were not assessed in this study. Further studies should be considered to address this limitation.

In conclusion, our findings demonstrate that patients with iCD exhibit atrophy in specific thalamic nuclei, particularly the intralaminar and ventral thalamic nuclei, highlighting the crucial role of the thalamus in the pathophysiology of iCD. The thalamus should not be considered as a single, homogeneous structure because of potentially useful information about distinct thalamic nuclei in future dystonia studies.

## Data Availability

The data and materials supporting the findings of this study are available from the corresponding author upon reasonable request.

## Abbreviations

BoNT: Botulinum toxin
BSP: Blepharospasm
CeM: Central medial
CM: Centromedian
FDR: False discovery rate
FHD: Focal hand dystonia
HAMA: Hamilton anxiety rating scale
HAMD: Hamilton depression rating scale
HCs: Healthy controls
iCD: Idiopathic cervical dystonia
LGN: Lateral geniculate
MGN: Medial geniculate
MMSE: Mini-Mental State Examination
MRI: Magnetic resonance imaging
MV(Re): Reuniens (medial ventral)
Pc: Paracentral
Pf: Parafascicular
Pt: Paratenial
SPSS: Statistical Package for the Social Sciences
STDT: Somatosensory temporal discrimination threshold
TWSTRS: Toronto Western Spasmodic Torticollis Rating Scale
VLp: Ventral lateral posterior
VM: Ventromedial
VPL: Ventral posterolateral
